# East and West Ethnography in Adult Day Services: Lessons From Taiwan and the United States

**DOI:** 10.1093/geroni/igaa057.1665

**Published:** 2020-12-16

**Authors:** Chih-ling Liou, Sonia Salari

**Affiliations:** 1 Kent State University at Stark, North Canton, Ohio, United States; 2 The University of Utah, Salt Lake City, Utah, United States

## Abstract

Adult Day Services (ADS), an alternative to institutionalization, offer daytime programs for those with physical and/or cognitive impairments. ADS have grown rapidly in Taiwan and the U.S., to improve quality of life (QOL) and provide respite for caregivers. ADS researchers have focused primarily on caregiver outcomes, with less emphasis on consumers. Our study is among the first to examine these issues in a cross-cultural context. Surveys are insufficient for ADS attendees, since many have diminished capacity. We use ethnographic methods to more fully understand the life of clients in ADS in two countries. The focus is on clients and staff, while including the physical and social environments. Data were collected over 820 hours of observation and 77 interviews with key ADS stakeholders, including clients, for insider perspective. Comparisons between the two countries, found that infantilization (e.g., baby-talk, child-oriented activities and decor) occurred in all 10 settings to varying degrees and outcomes were shaped by culture. In Taiwan, infantilization embedded within a cultural tradition of respecting elders and exhibited as a parent-child role reversal, where staff played the adult-child role. Their over-helping and over-controlling were aimed to ‘benefit’ clients, who were seen as incompetent aging parents. In the U.S., infantilization resembled a primary school environment with elementary décor. Aides used baby-talk and ‘taught’ compulsory child-oriented songs and games. This effect was exacerbated when preschool-aged children were added, and all generations were treated similarly. Results can be translated to implement age-appropriate care environments across cultures to improve QOL in ADS.

